# KCTD15 inhibits the Hedgehog pathway in Medulloblastoma cells by increasing protein levels of the oncosuppressor KCASH2

**DOI:** 10.1038/s41389-019-0175-6

**Published:** 2019-11-04

**Authors:** Eleonora Spiombi, Annapaola Angrisani, Simone Fonte, Giuseppina De Feudis, Francesca Fabretti, Danilo Cucchi, Mariapaola Izzo, Paola Infante, Evelina Miele, Agnese Po, Laura Di Magno, Roberto Magliozzi, Daniele Guardavaccaro, Marella Maroder, Gianluca Canettieri, Giuseppe Giannini, Elisabetta Ferretti, Alberto Gulino, Lucia Di Marcotullio, Marta Moretti, Enrico De Smaele

**Affiliations:** 1 Department of Molecular Medicine, Sapienza University of Rome, 00161 Rome, Italy; 2 Center for Life Nano Science@Sapienza, Istituto Italiano di Tecnologia, 00161 Rome, Italy; 3 Kymab ltd, Babraham Campus, Cambridge, CB22 3AT UK; 4 Department of Biotechnology, University of Verona, Verona, Italy; 5 Istituto Pasteur, Fondazione Cenci-Bolognetti, Sapienza University of Rome, 00161 Rome, Italy; 6 Department of Experimental Medicine, Sapienza University, 00161 Rome, Italy; 7 Present Address: Department of Medical Biotechnology and Translational Medicine, University of Milan, 20090 Segrate, Milan Italy; 8 Present Address: Department of Experimental Oncology, European Institute of Oncology, 20139 Milan, Italy; 9 Present Address: Barts Cancer Institute, Queen Mary University of London, Centre for Molecular Oncology, John Vane Science Center, London, EC1M 6BQ UK; 10 Present Address: Department of Hematology/Oncology and Stem Cell Transplantation, Bambino Gesù Children’s Hospital, IRCCS, 00165 Rome, Italy

**Keywords:** CNS cancer, Molecular biology

## Abstract

Medulloblastoma (MB) is the most common malignant childhood brain tumor. About 30% of all MBs belong to the I molecular subgroup, characterized by constitutive activation of the Sonic Hedgehog (Hh) pathway. The Hh pathway is involved in several fundamental processes during embryogenesis and in adult life and its deregulation may lead to cerebellar tumorigenesis. Indeed, Hh activity must be maintained via a complex network of activating and repressor signals. One of these repressor signals is KCASH2, belonging to the KCASH family of protein, which acts as negative regulators of the Hedgehog signaling pathway during cerebellar development and differentiation. KCASH2 leads HDAC1 to degradation, allowing hyperacetylation and inhibition of transcriptional activity of Gli1, the main effector of the Hh pathway. In turn, the KCASH2 loss leads to persistent Hh activity and eventually tumorigenesis. In order to better characterize the physiologic role and modulation mechanisms of KCASH2, we have searched through a proteomic approach for new KCASH2 interactors, identifying Potassium Channel Tetramerization Domain Containing 15 (KCTD15). KCTD15 is able to directly interact with KCASH2, through its BTB/POZ domain. This interaction leads to increase KCASH2 stability which implies a reduction of the Hh pathway activity and a reduction of Hh-dependent MB cells proliferation. Here we report the identification of KCTD15 as a novel player in the complex network of regulatory proteins, which modulate Hh pathway, this could be a promising new target for therapeutic approach against MB.

## Introduction

Medulloblastoma (MB) is the most common malignant brain tumor in children, representing nearly 20% of all childhood brain cancers^1^. Transcriptional and molecular characterization of MBs has led to their classification into four primary subgroups: (I) SHH-activated; (II) WNT-activated; (III) Group 3; and (IV) Group 4^2–4^. Group I MBs represents about 30% of all MBs and is characterized by constitutive activation of the Sonic Hedgehog (Hh) signaling pathway.

The Hh pathway is involved in fundamental processes during embryogenesis and in adult life, including cell proliferation, differentiation and morphogenesis, stem cell maintenance and regeneration^5,6^, but also in tumorigenesis.

During cerebellar development, Hh induces the growth of the external granule layer (EGL), which is mainly formed by Granule Cell Progenitors (GCPs)^7–9^. At the completion of the proliferative stage, Hh signal must be turned off, GCPs migrate to the cerebellar internal granule layer (IGL) and differentiate in mature neurons. If the Hh pathway remains accidentally turned on, it may lead to cerebellar tumorigenesis^10^.

Hh signaling pathway is typically initiated by the expression of Hh secreted ligands, which bind to the transmembrane receptor Patched1 (Ptch)^5^. In the absence of Hh, Ptch represses the other transmembrane receptor Smoothened (Smo) acting as a negative regulator of the pathway. In the presence of Hh, Ptch releases Smo which migrates to the cell primary cilium and leads to the activation of downstream transcription factors Gli1-3^5^. Non-canonical activation of the Hh pathway has also been detected in several tumors, due to ligand-independent signals, such as K-RAS signaling, TGFβ, PI3K, PKC, and epigenetic regulators, that act downstream of Smo, leading to the activation of Glis^11,12^. These observations, together with the partial failure of Smo inhibitors in treatment of a number of Hh-dependent tumors, highlight the need for therapeutic targeting of the pathway at the level of Gli transcription factors rather than at the level of the upstream receptors^11^.

KCASH2^KCTD21^ belongs to the family of protein KCASH (KCTD Containing-Cullin Adaptors, Suppressors of Hedgehog) and acts as negative regulator of Hh, inhibiting Gli transcription factors activity^13,14^. All three members of the family (KCASH1, KCASH2 and KCASH3) can form homo or hetero-oligomers and bind Cullin3 to form a E3 ubiquitin ligase complex, which recruits HDAC1 deacetylase inducing its degradation^14^. Since HDAC1 deacetylates the transcription factors Gli1 and Gli2 and enhances their transcriptional activity^14,15^, degradation of HDAC1 induced by the Cul3/KCASH complex prevents the activation of Gli1, inhibiting the Sonic Hedgehog pathway.

Genetic or epigenetic events that reduce the expression of KCASH family members allow the accumulation of HDAC1 and consequently the deacetylation of Gli1, that becomes fully transcriptionally active. Thus, the loss of KCASH may lead to persistent Hh activity and tumorigenesis^13^.

KCASH proteins act in concert to suppress the Hh pathway. However, the role of these proteins is not identical. For example, while KCASH1 and KCASH2 bind directly on HDAC1, KCASH3 cannot do so, but requires the formation of heterodimers with KCASH1. Indeed, while the three proteins have somewhat redundant functions, and share a good degree of homology at the N-term of the proteins, they may interact with additional different partners. Furthermore, they present a significant divergence in the C-terminal halves of the protein, suggesting the presence of peculiar functions or different mechanisms of individual KCASHs functional regulation.

In order to better characterize the physiologic role and mechanisms of KCASH2 modulation, we have searched by means of a proteomic approach for new KCASH2 interactors, identifying Potassium Channel Tetramerization Domain Containing 15 (KCTD15).

Here we identify a new role for KCTD15 on Hh pathway suppression, defining its function as a stabilizer of KCASH2, which increases its inhibitory activity.

## Results

### Identification of KCTD15 as a new KCASH2 interactor

To identify potential interactors of KCASH2 we searched interacting proteins in HEK293T cells transfected with an expression vector encoding human KCASH2, N-terminally HA and Flag tagged (Fig. S1A), performing a sequential double co-immunoprecipitation (Co-IP) of the cell lysate. The co-immunoprecipitated proteins were then analyzed by mass spectrometry analysis and polypeptides that were represented with a number of peptides ≥3 were selected for further analysis (Fig. S1B).

The most represented interactor was KCTD15.

Similarly to KCASH2, KCTD15 is a member of the KCTD family of proteins and contains a conserved BTB domain (characteristic of all the KCTD family members). Human KCTD15 gene maps to chromosome 19q13.11 and encodes a 283 amino acids (31 KDa) protein.

KCTD15 function has been described mostly in neural crest^16^, although other data indicate that it is expressed also in different adult mouse and human tissues, including adult brain and cerebellum^17–19^ suggesting a potential interplay with KCASH2, which has been described to play an important role in Hh regulation during cerebellar development and differentiation^13^.

**Fig. 1:**
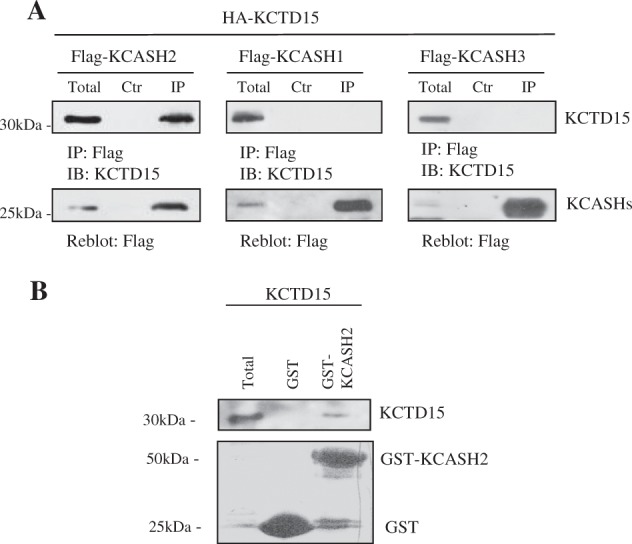
KCTD15 interacts specifically with KCASH2. **a** KCTD15 co-immunoprecipitates with KCASH2 but not with KCASH1 and KCASH3. Co-IP assays was performed on total lysates from HEK293T cells transfected with expression vectors encoding for the indicated proteins and immunoprecipitated (IP) with anti-Flag agarose beads. IP samples and a fraction of the total lysate (Total) were separated on SDS-PAGE gels. Blots were immunoblotted (IB) with anti-KCTD15 antibody and reblotted with anti-Flag antibody. **b** KCASH2 interacts with KCTD15 in vitro. IVT KCTD15 was incubated with GST control or GST-KCASH2. After GST pull-down (see lower panel), the interacting protein was detected by WB with anti-KCTD15 antibody

To validate the mass spectrometry data, we performed Co-IP experiments following overexpression of HA-tagged KCTD15 and Flag-tagged KCASH2 in HEK293T cells, thus confirming the interaction of the two proteins (Fig. 1a, left panel). Because of the high homology within the KCASH proteins and their shared functions, we also evaluated if KCTD15 interacts with the other KCASH proteins: KCASH1 and KCASH3. As shown in Fig. 1a (central and right panels), KCTD15 does not coimmunoprecipitate with KCASH1 and KCASH3, indicating a specific interaction of KCTD15 with the KCASH2 family member. We next verified that KCASH2-KCTD15 interaction does not require the presence of further adaptor proteins, since a GST pull-down assay performed with in vitro translated (IVT) KCTD15 and KCASH2-GST fusion protein indicates that KCTD15 can bind to GST-KCASH2, while does not bind to GST alone control (Fig. 1b).

We next investigated whether the two proteins can be present in the same subcellular compartments. To this end, we expressed KCTD15 and KCASH2 in the MB cell line DAOY^20^ and observed that KCASH2 and KCTD15 indeed colocalize both in the cytoplasm and in the nuclei, although KCTD15 seems to have a relatively stronger staining in the nuclei (Fig. S2). This latter observation, in which KCASH2 expression does not mirror exactly the KCTD15 expression, suggests that KCTD15 colocalizes with KCASH2 but does not sequester or relocate the KCASH2 protein.

### KCTD15 can form homo-oligomeric and hetero-oligomeric complexes and interacts with KCASH2 through the BTB/POZ domain

The BTB/POZ domain is a protein-protein interaction domain involved in oligomerization of many KCTD family members^21^. Indeed, the KCASH proteins may form homo-oligomers and hetero-oligomers through their BTB/POZ domains^13^.

**Fig. 2:**
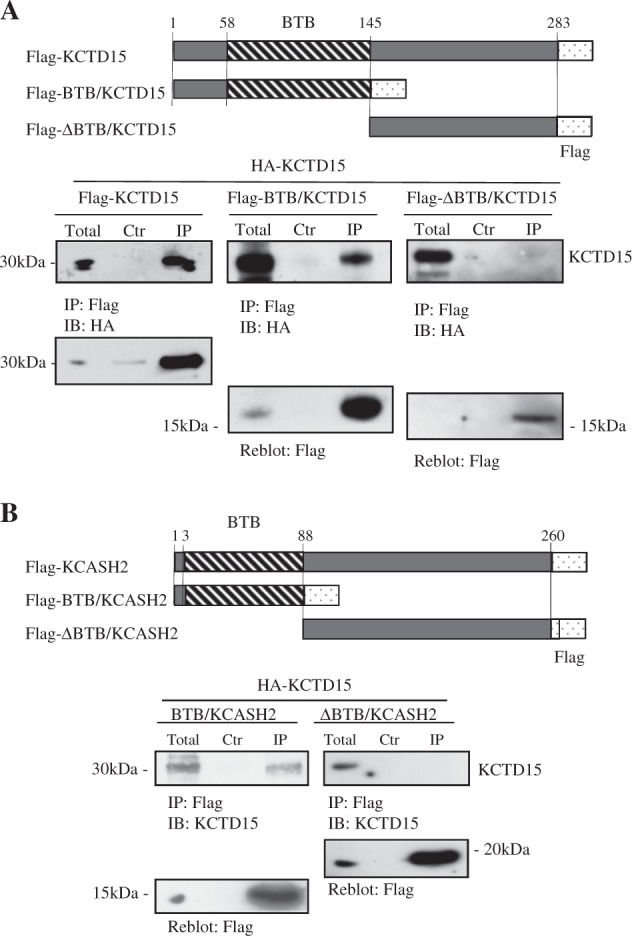
KCTD15 homo-and hetero-oligomerizes via BTB-POZ domain. **a** KCTD15 forms homo-oligomeric complexes via the BTB-POZ domain. Co-IP experiments performed in lysates from HEK293T cells cotransfected with expression vectors encoding for the indicated proteins. IP was performed with anti-Flag conjugated agarose beads. Blots were IB with anti-HA antibody and reblotted with anti-Flag antibody. **b** KCTD15 forms hetero-oligomeric complexes with KCASH2 via the BTB-POZ domain. Co-IP experiments were performed as above after co-transfection of vectors expressing KCTD15-HA or KCASH2 fragments as depicted in the upper panel. IP was performed with anti-Flag agarose beads, IB with anti-KCTD15 antibody and IP was controlled by reblot with anti-Flag antibody

Here, by expressing the full-length KCTD15, the BTB-containing N-terminal half of the protein (BTB-KCTD15) and the C-terminal half lacking the BTB domain, (called ΔBTB-KCTD15; see Fig. 2a upper panels) followed by immunoprecipitation, we demonstrated that KCTD15 is able to form homo-oligomeric complexes only when the BTB domain is present, suggesting that BTB is the actual oligomerization domain (Fig. 2a).

Similarly, we tested if KCTD15 interacts with the BTB domain of KCASH2. To this purpose, we expressed BTB-KCASH2 or the KCASH2 C-terminal region (ΔBTB-KCASH2; see Fig. 2b upper panel) in HEK293T cells. Following immunoprecipitation assay and Western Blot analysis, we demonstrated that KCTD15 coimmunoprecipitates with the BTB-containing KCASH2 but it does not with the ΔBTB fragment (Fig. 2b lower panels), indicating that BTB domains mediate the interaction of the two proteins.

### KCTD15 downregulates Gli1 transcriptional activity and HDCA1 protein levels but does not bind neither Cul3 nor HDAC1

Although KCTD15 has been abscribed to a distinct subgroup of KCTD proteins, it shares a good degree of similarity in the BTB domain with the KCASH family of proteins^22,23^. This observation, together with the physical interaction with KCASH2, suggests that these proteins might act together and share functional activity.

KCASH2 negatively regulates the Hh signaling, promoting HDAC1 degradation, increasing Gli1 acetylation and consequently suppressing its transcriptional function^13,14^.

We investigated whether KCTD15 was able to control Hh pathway, acting through a similar mechanism. We performed a preliminary screening on a group of 47 human sporadic MB samples and nine cerebellar controls, by measuring the expression levels of KCTD15. Interestingly, in a relevant subset of the SHH subgroup (5/19, 26.3% of the samples) there was a marked reduction of KCTD15 mRNA levels (Fig. S3a), supporting a role for KCTD15 in Hh context. It has to be noted that among the other subgroups, only 2/14 samples in Group 3 (which overall presents higher KCTD15 expression) presented a KCTD15 reduction.

Analyzing the same samples for the KCASH2 expression levels, we confirmed that KCASH2 was reduced in most of SHH MBs, as previously described^13^ (Fig. S3b). Of note, we could not find a correlation between expression levels of KCTD15 and KCASH2 in this context (Fig. S3c), indicating that KCTD15 is not acting on KCASH2 transcription.

**Fig. 3:**
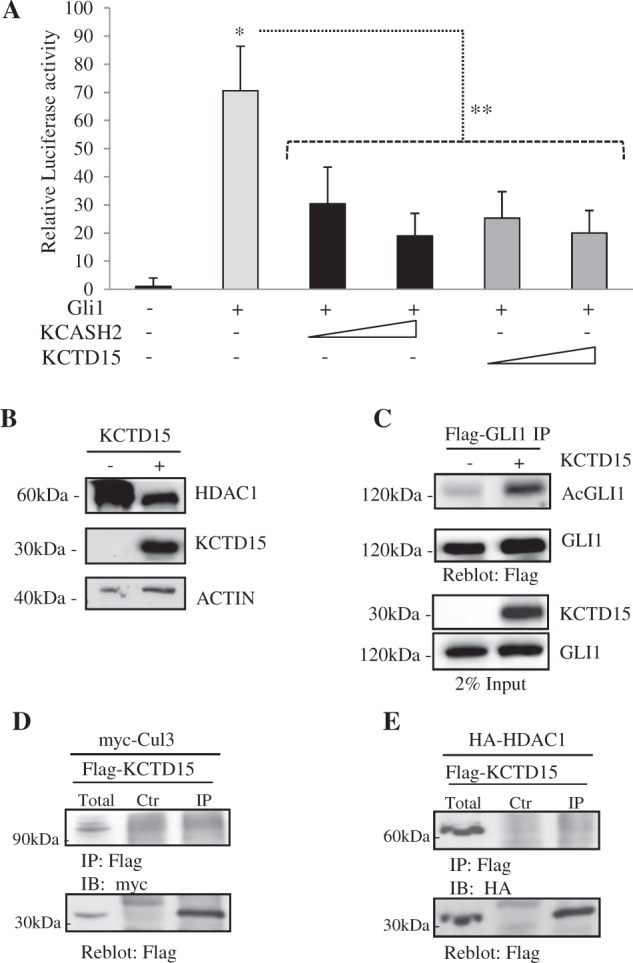
KCTD15 reduces Gli1 transcriptional activity and HDAC1 protein levels but does not interact with Cul3 and HDAC1. **a** KCTD15 expression reduces Gli1-responsive Luciferase activity. Assays were performed on lysates from HEK293T cells transfected with 12×-GliRE-Luc alone or with Gli1, KCASH2, KCTD15 and pRL-TK Renilla (as a normalizer). Data are indicated as mean ratios with respect to Renilla luciferase signal. *P < 0.05 Gli1 versus control, **P < 0.01 KCASH2 or KCTD15 versus Gli1. **b** KCTD15 expression reduces HDAC1 protein levels. HEK293T were co-transfected with HDAC1-HA vector, plus empty or Flag-KCTD15 vectors and protein lysates were analyzed by WB. **c** HEK293T were co-transfected with Flag-GLI1 vector plus empty or KCTD15. IP was performed with anti-Flag conjugated agarose beads. Blots were IB with anti-acetylated lysine (K518) antibody and reblotted with anti-Flag antibody (upper panel). Total lysates were analysed by Western Blot using anti-KCTD15 and anti-Flag antibodies (lower panel). **d**, **e** KCTD15 is not able to interact with Cul3 (**d**) and HDAC1 (**e**). Co-IP experiments are performed in lysates from HEK293T cells transfected with expression vectors encoding for the indicated proteins

Overexpression of KCTD15 in HEK293T cells significantly decreases the transcriptional activity of Gli1-responsive luciferase reporter (Fig. 3a). To further investigate KCTD15 mechanism of action, we evaluated if KCTD15 overexpression acted on Gli1 by affecting the levels of HDAC1, and indeed observed a significant reduction of HDAC1 protein (Fig. 3b). Moreover, we monitored the levels of Gli1 acetylation following KCTD15 overexpression, confirming that HDAC1 degradation leads also to a decrease in HDAC1 activity (Fig. 3c).

Next, we wondered if the activity of KCTD15 on HDAC1 was mediated by the formation of a complex recruiting Cul3 on HDAC1 or by a different mechanism. Indeed a previous report, based on data obtained using recombinant proteins expressed in a bacterial system, suggested that KCTD15 may be unable to bind Cul3^23^.

By performing a co-immunoprecipitation experiment with KCTD15, we ruled out an interaction between KCTD15 and Cul3 or HDAC1 (Fig. 3d, e).

These data suggest an indirect mechanism of action for KCTD15 on the Hh pathway, and led us to verify if the action of KCTD15 could be mediated by an effect on KCASH2.

### KCTD15 increases KCASH2 protein stability, thus enhancing its activity on Gli1

We hypothesized that KCTD15 expression may lead to an increased KCASH2 protein activity or stability.

**Fig. 4:**
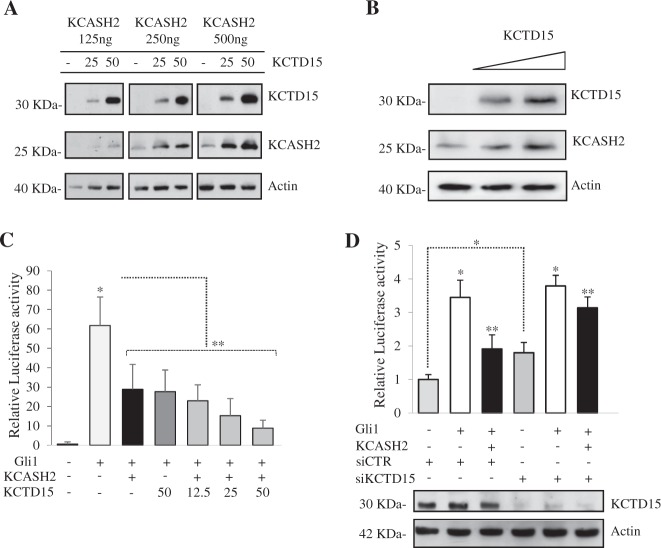
KCTD15 increases KCASH2 protein levels, enhancing its inhibitory activity on Gli1. **a**, **b** Exogenous and endogenous KCASH2 protein levels increase in presence of KCTD15. **a** HEK293T cells were transfected with different amounts of Flag tagged KCTD15 and KCASH2 (as indicated), then lysed and analyzed by WB with anti-Flag antibody. Actin protein is shown as a normalizer. **b** HEK293T cells were transfected with increasing amount of KCTD15-Flag and endogenous KCASH2 was detected by anti-KCASH2 antibody. Actin was used as a loading control. **c** KCTD15 increases the inhibition of GliRE-luciferase activity in KCASH2 co-transfected cells. HEK293T cells were transfected with 12×GliRE-Luc and pRL-TK Renilla (as a normalizer) plus variable amounts of the indicated vectors. *p < 0.05 versus control. **p < 0.05 versus Gli1. **d** Depletion of endogenous KCTD15 reduces KCASH2 inhibitory activity on Gli1. Relative luciferase activity was measured in HEK293T cells transfected with scrambled siRNA (siCtr) or with KCTD15 siRNA (siKCTD15) followed by 12×GliRE-Luc and pRL-TK Renilla plus the indicated vectors. Bottom panel shows KCTD15 protein levels. Actin was used as loading control. *p < 0.05 *versus* control. **p < 0.05 *versus* Gli1 transfected

Indeed, overexpressed KCTD15 increases the protein levels of both exogenous (Fig. 4a) and endogenous (Fig. 4b) KCASH2 in HEK293T cells dose-dependent manner, leading to the hypothesis that its activity on Hh pathway is an effect of the increase of KCASH2 protein in the cells.

We tested this hypothesis by performing a Gli-responsive luciferase reporter assay in which we co-transfected different amounts of KCTD15 alone or together with a fixed amount of KCASH2 expressing vector. As shown in Fig. 4c, KCTD15 increases the inhibition of GliRE-luciferase activity in KCASH2 co-transfected cells in a dose-dependent manner. Furthermore, KCTD15 alone has an inhibitory effect that can be related to stabilization of the endogenous KCASH2.

To verify if the presence of KCTD15 has a physiologically relevant function, we monitored the effect of the depletion of endogenous KCTD15 in HEK293T cells on a GliRE-luciferase assay. As expected, siRNA-mediated depletion of endogenous KCTD15 (Fig. 4d, lower panel) abrogated its stabilizing effect on KCASH2, increasing the baseline of Gli1 transcriptional levels (See Fig. 4d, fourth column), and reducing the inhibitory efficiency of KCASH2 overexpression (Fig. 4d, sixth column). Next, we confirmed that KCTD15 ability to inhibit Gli1 activity depends on the presence of KCASH2. To this end, we silenced KCASH2 expression in HEK293T cells (Fig. S4) and performed in these cells a GliRE-luciferase assay following overexpression of KCTD15. Indeed, KCTD15 suppressive activity resulted abolished (Fig. S5).

### KCTD15 expression increases KCASH2 protein levels and reduces Hh-dependent medulloblastoma cells proliferation

**Fig. 5:**
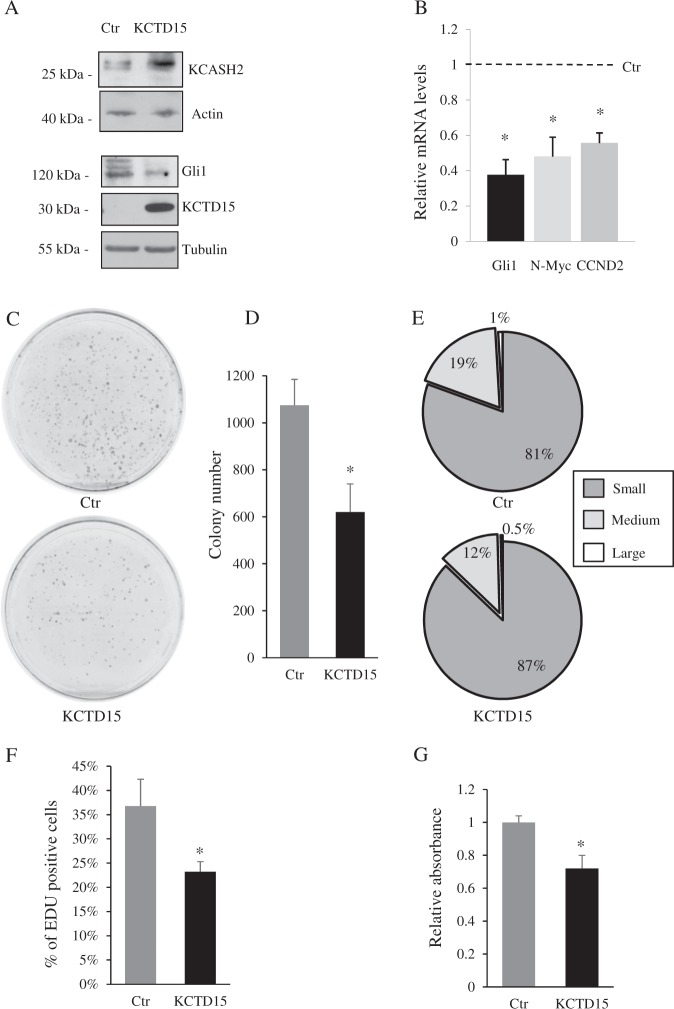
KCTD15 expression increases KCASH2 protein levels, and reduces Hh-dependent medulloblastoma cells proliferation. **a** KCASH2 protein levels are increased in DAOY MB cells expressing KCTD15 while Gli1 protein is reduced. DAOY cells were transfected with KCTD15-Flag and protein lysates were immunoblotted with anti-KCASH2 antibody (upper panels) or anti-Gli1, anti-Flag antibodies (lower panels). Anti-Actin and anti-Tubulin antibodies were used as loading controls. **b** Hh pathway activity is downregulated in KCTD15-transfected MB cells. Q-RT-PCR analysis of endogenous Hh targets mRNA levels are normalized to the control (Ctr). *p < 0.05 versus Ctr. **c**–**g** MB cells proliferation is reduced following KCTD15 expression. DAOY cells overexpressing KCTD15 were assayed for their capability to form colonies (**c–e**). Representative images were shown from three independent experiments (**c**) and number of colonies (**d**) are shown in correlation with the empty vector (Ctr). **e** Percentage of small, medium, and large size colonies is indicated in DAOY Ctr cells (upper panel) and DAOY transfected with KCTD15 (lower panel). **f** DAOY cells were transfected with indicated vectors for 24 h. Following 6 h incubation with EdU, the cells were fixed and stained with Click-iT kit. Number of EdU positive cells was calculated over total cells and expressed as percentage vs total cells. Results are expressed as the mean ± SD of three independent experiments (*P < 0.05, Student’s *t*-test). **g** MTT assay on DAOY cells transfected with empty or KCTD15 vectors and analyzed after 24 h. Results are expressed as the mean ± SD of five independent experiments, each performed in triplicate (*P < 0.05, Student’s *t*-test)

KCASH2 has been previously shown to suppress medulloblastoma cell line DAOY growth by negatively regulating Hh/Gli1 signaling^13^. To verify the effect of KCTD15 on this tumor model, we overexpressed KCTD15 in DAOY cells. As expected, overexpression of KCTD15 led to an increase of endogenous KCASH2 protein levels (Fig. 5a), and a concomitant reduction in Hh activity, measured both by monitoring Gli1 protein levels (Fig. 5a) and expression of Hh target genes, such as Gli1, N-myc, CyclinD2 (CCND2)^24^ (Fig. 5b).

Accordingly, colony assays performed on control and KCTD15 overexpressing DAOY cells demonstrated a reduced Hh-dependent proliferation (Fig. 5c, d).

Interestingly, not only the absolute number of the colonies was reduced by 40%, but also the size distribution of the colonies was affected (Fig. 5e). Indeed, we observed a marked reduction in the percentages of the medium (from 19 to 12%) and large (from 1 to 0.5%) colonies in KCTD15-transfected cells. This suggests that the overexpression of KCTD15 leads to both a reduced Hh-dependent stemness of MB cells (which is indicated by a reduced number of colonies) but also a reduction of the proliferative potential of the cells, which leads to smaller colonies. This is consistent with Hh role in tumorigenesis, which is known to affect both proliferation and cancer cell stemness^6,25,26^.

To confirm the anti-proliferative effect of KCTD15 on DAOY cells, we performed an EdU incorporation assay to detect and quantify cell proliferation using fluorescence microscopy. As shown in Fig. 5f and Fig. S6, cells overexpressing KCTD15 presented a reduced percentage of EdU positivity. Similar results were obtained using an MTT assay (Fig. 5g). While FACS analysis of KCTD15 overexpressing cells did not highlight significant alterations in cell cycle (not shown), evaluation of apoptosis revealed a small but significant difference in the apoptotic rate of KCTD15-transfected cells (Fig. S7a and S7b). Apoptosis was detected through DNS assay (Differential Nuclear Staining)^27^ and analyzed by fluorescence microscopy, revealing a doubling in apoptotic cells (from 2.5 to 5%) in KCTD15 overexpressing cells (Fig. S7a). Western Blot analysis for cleaved Caspase3 confirmed this result (Fig. S7b).

## Discussion

Modulation of the Hh signaling pathway is critical for proper cerebellar development, and aberrant Hh activity is responsible for a large fraction of all human MB. We have previously identified a family of Hh suppressors, the KCASH family, which acts by ubiquitylation and degradation of the HDAC1 deacetylase, leading to Gli1 acetylation and repression of its transcriptional activity.

Here we report the identification of KCTD15 as a novel player in the complex network of regulatory proteins which modulate Hh pathway. Indeed, KCTD15 is able to interact with the Hh inhibitor KCASH2, stabilizing the protein and enhancing its inhibitory effect in MB cells.

Our preliminary analysis of expression on a group of MB samples (Supplementary Fig. 3) has suggested that low levels of KCTD15 may be present in SHH group MB. Indeed, these results, although suggestive, indicate that only a subpopulation of SHH medulloblastoma expresses significantly low KCTD15 mRNA levels. It is possible that a certain number of MB may express mutated forms of KCTD15 or have downstream alterations in KCASH2 (loss of expression, mutations) or Hh pathway components that render not necessary KCTD15 suppression. Indeed, statistical analysis performed on a larger group of samples in publicly available database^28^ confirmed that, considering a wider cohort of SHH MB tumors, there is no significant correlation with KCTD15 expression (not shown). This may be due in part to the fact that KCTD15 is not a direct modulator of the Hh pathway, but acts through the modulation of one inhibitor that is KCASH2, adding complexity and background noise to the analysis. Furthermore, it is likely that tumors that have already a significant reduction of KCASH2 expression (KCASH2 low, very frequent in the SHH group), may not be dependent on a reduction of KCTD15 expression, and this may lead to loss of statistical relevance of KCTD15 reduction in this group.

It must be considered, furthermore, that KCTD15 action is not through modulation of KCASH2 mRNA expression, and for this reason it cannot be expected a positive correlation between KCASH2 and KCTD15 mRNA expression. In fact, in our in vitro experiments, we never observed any effect of KCTD15 overexpression or downmodulation on KCASH2 mRNA levels (not shown).

Coherently, we did not find in our SHH MB samples (and in publicly available databases) a direct correlation between KCASH2 and KCTD15 mRNA expression (Supplementary Fig. 3c, *R* = 0.1, *p* = 0.69; and not shown).

KCTD15 protein has been previously grouped together with KCTD1 in a clade of the KCTD family which has been attributed non-protein degradation functions^22^. In fact, KCTD15 does not bind Cul3 and thus does not appear to be able to directly ubiquitinate and degrade its protein interactors. Indeed, in our context, interaction of KCTD15 with KCASH2 leads to KCASH2 protein stabilization, which could be hypothesized to be due to a conformational change of the oligomer, which may protect it from faster protein turn-over.

Further work will be needed to explore the conformations acquired by the KCASH2-KCTD15 oligomers, since their interaction does not seem to affect negatively the capability of KCASH2 to bind Cul3 (data not shown) and definitely does not affect negatively its inhibitory role on the Hh pathway transcriptional activity.

KCTD15 has been previously credited with transcriptional repression functions^22^. This has been demonstrated in vertebrate neural crest during development, in which KCTD15 has been demonstrated to negatively regulate the transcription factor TFAP2A, a target of WNT signaling^16^ by binding to it, changing its conformation and precluding transcriptional activation^16^.

KCTD15 function on TFAP2A seems mostly restricted to neural crest delimitation during the embryonal developmental phase^16^. It has also been noted that expression pattern of TFAP2 and KCTD15 are not largely overlapping^16^ and therefore KCTD15 necessarily plays other roles in different tissues.

KCTD15 expression has been previously reported in adult cerebellum^19^. Interestingly, most of the cerebellar cell types (including cerebellar granule neuron, Purkinje cells, Bergmann Glia, astrocytes) do not derive from neural crest but from the neural tube, and TFAP2A expression has been reported to be limited to developing and adult cerebellar GABAergic interneurons (Purkinje cells;^29^). Thus, KCTD15 most probably plays different functions in cerebellar context, one of whom is the modulation of KCASH2 in Hh-responsive cells (i.e. cerebellar granule cells).

Here, we demonstrate that KCTD15 contributes to Hh signaling control, through stabilization of KCASH2. Indeed, overexpression of KCTD15 in MB cell line DAOY induces suppression of Hh signaling and reduction of cell proliferation.

Since modulation of Hh pathway may be also mediated by effects on Hh pathway proteins trafficking in the primary cilium, a specialized sub-cellular site that coordinates Hh signal transduction, we verified by overexpressing tagged proteins in DAOY cells that KCTD15 and KCASH2 are not localized in the cilium (Fig. S8).

We hypothesize that loss (or reduction) of KCTD15 expression in cerebellum may lead to a reduced KCASH2 stability and to an incomplete turn-off of the Hh pathway, with a consequent increased proliferation of GCPs, delayed differentiation and tumorigenesis. Interestingly, our preliminary screening on sporadic human MBs has revealed a marked reduction of KCTD15 expression levels in a subset of the Hh subgroup (Fig. 3a). Of note, the WNT group expressed high levels of KCTD15, suggesting the possibility that in this context KCTD15 may act again by suppressing TFAP2A transcription and consequently its potential WNT/beta-catenin inhibition^30^. The identification of this new function of KCTD15 does not rules out further roles for KCTD15 in other tissues, since both the presence of the KCTD domain suggest the potential for further protein-protein interactions, and the described transcriptional repression activity may be effective not only on AP2a but also on other transcription factors.

The new role identified for KCTD15 suggests novel potential therapeutical approaches for modulating the Hh pathway in Hh dependent tumors. Indeed, the use of HDAC inhibitors has been suggest by several group as a potential therapeutical approach^31^, alternative to the resistance-prone Smo inhibitors^32^ although the spectrum of potential HDAC targets in cells does not allow to exclude unwanted side effects. Up to date there are no available strategies to positively regulate KCTD15 expression in cancer, though further studies on its transcriptional modulation may uncover potential pharmacological modulators. A future strategy may be to use bioinformatic, proteomic and biochemical approaches to identify KCTD15/KCASH2 critical binding sites and generate small peptides or small molecules that may mimic KCTD15, binding and stabilizing KCASH2. This stabilization would in turn increase HDAC1 degradation switching off Hh signaling. This approach may be the easiest therapeutical strategy to implement the KCASH2 inhibitory function in vivo, given that other strategies such as overexpression of exogenous KCASH2 in tumor cells may be more technically challenging.

## Materials and methods

### Human tissue samples

Human primary MB specimens were collected by surgical resections with the approval of Sapienza University institutional review board as previously described^13^. Informed consent was obtained from all subjects. RNAs of normal human cerebella were purchased from Biocat (Heidelberg, Germany), Ambion (Applied Biosystems, Foster City, CA), and BD Biosciences (San Jose, CA).

### Cell Culture, transfections, and luciferase assay

Medulloblastoma cell line DAOY (ATCC HTB-186) was cultured in minimum essential medium (Gibco- Thermo Fisher Scientific, Waltham, MA, USA) supplemented with 10% heat-inactivated fetal bovine serum (FBS), 1% Sodium pyruvate, 1% non-essential amino acid solution, 1% l-glutamine and 1% penicillin/streptomycin. DAOY cells were transfected with Lipofectamine Plus, according to the manufacturer’s instructions (Invitrogen- Thermo Fisher Scientific). For primary cilium analysis, cells were kept in low serum (0.5% FBS) 48 h to promote cilia formation^33^.

HEK293T cells (ATCC CRL-3216) were cultured in Dulbecco Modified Eagle Medium (Gibco) supplemented with 10% FBS, 1% l-glutamine and 1% penicillin/streptomycin. HEK293T cells were transfected with Lipofectamine 2000, according to the manufacturer’s instructions (Invitrogen).

Mycoplasma contamination in cells cultures was routinely screened by using PCR detection kit (Applied Biological Materials, Richmond, BC, Canada).

KCTD15 RNA interference (siRNA) was performed using *Silencer*® Select Pre-designed siRNA (30 nM; 4392420, Ambion- Thermo Fisher Scientific), transfected with Lipofectamine 2000 according to the manufacturer’s instructions.

Luciferase and Renilla activity was assayed with a dual-luciferase assay system, following manufacturer’s instructions (Promega, Madison, WI, USA). Results are expressed as Luciferase/ Renilla ratios and represent the means ± SD of at least five biological replicates.

### Plasmids

The following vectors, expressing full length or truncated forms of human KCTD15 or KCASHs, with or without tags: pCDNA3.1-KCTD15; pCXN2-KCTD15, pCXN2-BTB-KCTD15, pCXN2-ΔBTB-KCTD15, tagged with Flag or HA; pCXN2-Flag-GLI1, pGEX-KCASH2, pCDNA3.1-HA-FLAG-KCASH2, pCXN2-KCASH1-Flag, pCXN2-KCASH3-Flag, pCXN2-KCASH2-Flag, pCXN2-BTB-KCASH2-Flag, pCXN2-ΔBTB-KCASH2-Flag were generated in our laboratory with standard cloning techniques and verified by sequencing. pEGFP-N1 was obtained from Clontech (Takara, Saint-Germain-en-Laye, France).

The following plasmids were kindly provided by other laboratories: 12X Gli-Luc and pCMV-SuFu (R. Tofgard, Karolinska Institutet, Sweden), pCMV-HDAC1 (P.L. Puri, The Burnham Institute, CA), pCDNA Cul3-myc (M. Pagano, New York University School of Medicine, NY).

The KCASH2 interference was performed using PLK0.1 plasmid expressing a specific shRNA (Mission shRNA-TRCN0000139768), and ShC002V as a control (SIGMA).

### Lentivirus production and transduction of target cells

Sub confluent HEK293T cells were cotransfected using Calcium/Phosphate precipitation, with lentiviral constructs shKCASH2 and shC002. Viral titers in supernatants were determined by MOI assay. The HEK293T cells were transduced with 1 × 10^6^ recombinant lentivirus-transducing units.

### MALDI-TOF mass spectrometry

Sub-confluent HEK293T cells were transfected by standard Calcium/Phosphate precipitation, with the Flag-HA-KCASH2 construct described above. Forty-eight hours after transfection cells were collected and lysed, then MALDI-TOF mass spectrometry was performed as previously described^34^.

### RNA isolation and quantitative real-time PCR

Total RNA from cells and tissues samples was extracted using TRIzol reagent, and cDNA synthesis was performed using the High Capacity cDNA reverse transcription kit (Invitrogen) according to the manufacturer’s instructions. Quantitative real-time PCR analysis of *Gli1, N-myc, cyclin-D2, KCTD15, B-actin, GAPDH,* and *HPRT* messenger RNA (mRNA) was performed on cDNAs employing using TaqMan gene expression assay according to the manufacturer’s instructions (Applied Biosystem- Thermo Fisher Scientific) and using the ViiA™ 7 Real-Time PCR System (Applied Biosystem). Experiments were replicated biologically at least 3 times, with 3 technical replicates each. All values were normalized to the endogenous controls *GAPDH, HPRT and B-actin*. mRNA quantification was expressed, in arbitrary units, as the ratio of the sample quantity to the mean values of control samples.

### Western blot analysis and immunoprecipitation

Cells were lysed with buffer containing: 50 mM Tris-HCl (pH 7.6), 1% deoxycholic acid sodium salt, 150 mM NaCl, 1% NP40, 5 mM EDTA, 100 mM NaF, and protease inhibitors. Total protein extracts were then separated on a denaturing SDS-PAGE gel and evaluated by Western blot assay using the antibodies listed below. For co-immunoprecipitation, lysates were incubated with agarose-conjugated Flag M2 beads (A2220; Sigma Aldrich-Merck, Darmstadt, Germany) for 2 h at 4 °C. Control sample antibody was saturated with anti-Flag peptide (F3290; Sigma). Beads were washed extensively with lysis buffer, and the complexes were evaluated by Western blot analysis. Antibody sources were as follows: mouse anti-tubulin polyclonal (SC-8035; Santa Cruz Biotechnology, Heidelberg, Germany) and mouse monoclonal anti-beta actin (A5441; Sigma) goat anti-actin polyclonal (SC-8432; Santa Cruz Biotechnology); mouse anti-HA monoclonal (H6533; Sigma), mouse anti-Flag-M2 (A8592; Sigma), rabbit anti-Myc (A5598; Sigma); rabbit anti-KCTD15 polyclonal antibody (20128-1-AP; Proteintech, Manchester, UK); anti-Gli1 mouse monoclonal (2643; Cell Signaling Technology, Danvers, MA, USA); rabbit anti-cleaved Caspase3 (9661S; Cell Signaling); rabbit anti-KCTD21/KCASH2 monoclonal (AB192259; Abcam, Cambridge, UK) Anti-KCASH2 mouse monoclonal was generated in house and described in [5]. Anti-acetylated Gli1 (K518) antibody rabbit polyclonal was previously described^15^. Secondary antibody anti–mouse (SC-516102) /rabbit (SC-2357)/goat IgG (SC-2354) conjugated with HRP were from Santa Cruz. Blots shown are representative of at least three independent experiments.

### GST pull down assay and in vitro translation

pGEX and pGEX-KCASH2 were expressed in BL-21-competent bacterial cells. The resultant GST-fusion proteins were purified using a glutathione column. Protein purity was assessed by SDS-PAGE and Comassie staining. In vitro translation reactions were performed following the manufacturer’s instructions (Promega). The reaction mix contains TNT Rabbit Reticulocyte, TNT Reaction Buffer, TNT RNA polymerase, Transcent Biotin-Lysyl-tRNA, amino acid minus methionine, Methionine 1 mM, RnasiOUT, and KCTD15-FLAG in PCDNA plasmid. This reaction was incubated at 30 °C for 90 min, followed by a luciferase reaction with standard luciferase assay (Biotium, CA) and control luciferase DNA (0.5 ug/ul) as a control. The IVT samples was, then, blotted on membrane and revealed by Streptavidin-HRP antibody (Promega).

GST-pull down experiment was performed in triplicate (biological replicates). Briefly, 100 mg of purified recombinant GST-KCASH2 was incubated with equal amount of GST protein in binding buffer (20 mM Hepes pH 7.9, 150 mM NaCl, 0.5 mM EDTA, 10% glycerol, 0.1% Tween 20 and a cocktail of protease inhibitor and PMSF) and incubated with 300 μl of glutathione-Sepharose 4B (Amersham-Sigma) at 4° for 2 h. Then, the beads were washed five times with the binding buffer, resuspended in Laemmli sample buffer and boiled. Pulled down proteins were resolved on SDS-polyacrylamide gels and revealed by western blot.

### Colony assay

DAOY MB cells were transfected as indicated above, then plated in 10 cm-diameter dishes (1 × 104 cells per dish) and after 2 weeks of neomycin selection, as previously described^13^, cell colonies were fixed and counted following crystal violet staining in 20% methanol. The number and size of the colonies were measured using the ImageJ plugins “colony-counter” (version 1.49v, Java 1.8.0_45, Wayen Rasband, U.S. National Institutes of Health, Bethesda, MD, USA; website: http://rsb.info.nih.gov/ij/downl oad.html). The colonies were splitted in small (range 1–49 points), medium (range 50–99 points), and large (≥100 points) (1 points = 1 pixel = 0,03 mm). Experiment was performed in five biological replicates for each condition.

### Proliferation assay

Click-iT™ EdU Alexa Fluor™ 488 Imaging Kit (Life Technologies, C10337) is optimized to label proliferating cells, in accordance with manufacturer’s instructions.

For MTT assay, cells were plated at a density of 9,000 cells/well in 96-well plates containing complete medium. Cell proliferation was detected using CellTiter 96 Aqueous One Solution (Promega) at 24 h following seeding. For cell proliferation measurement, 20 µl methanethiosulfonate reagent was added to the medium and incubated at 37˚C in a humidified 5% CO2 atmosphere. The absorbance was read at 490 nm.

### Flow cytometry analysis

The cells were harvested, fixed in chilled 70% ethanol, treated with RNase A (100 μg/ml RNase) for 15 min at 37 °C and stained with propidium iodine (PI) (10 μg/ml PI) as described^35^. Data acquisition and analysis was carried out using a flow cytometry. Data analysis was performed with CellQuestPro software (BD Biosciences, Milan, Italy).

### Immunofluorescence and DNS assay

DAOY MB cells were cultured on glass coverslips (chamber 8) and transfected with the indicated plasmids. After 24 h, cells were fixed, permeabilized and stained, as described^36^. Antibodies were as follows: mouse anti-HA (MMS-101R; Covance, NJ, USA) to identify KCASH2-HA and rabbit anti-Flag (F7425; Sigma) to identify KCTD15-Flag; rabbit anti-ARL13B (17711-1-AP, Proteintech, Rosemont, USA) to stain the cilia. Specific signals were revealed by AlexaFluor 488 (A11001) and 594 (A21203) secondary antibodies, respectively (Life Technologies). The nuclei were counterstained with Hoechst 33342 (U0334; Abnova, Germany). For DNS assay, DAOY cells were cultured and transfected with indicated plasmids. After 48 h, cells were incubated with propidium iodide (10 μM, Sigma P4864, PI) and Hoechst 33342 (10 μM) for 10 min, as described^27^. Images were acquired on a LEICA DM 2000 microscope.

### Statistical analysis

For all luciferase, QPCR, MTT, proliferation, and apoptotic assays, colony formation assay experiments, the *P* values were determined using Student’s t-test and statistical significance was set at *P* < 0.05 or *P* < 0.01. Data were assumed to have normal distribution. Results are expressed as mean ± S.D. *P* values for MB samples was calculated by Mann Whitney test. All experiments presented were representative of at least five biological replicas, except when specifically indicated. Correlation analysis was measured using GraphPad Prism 6 software (La Jolla, CA, USA), as previously described^37^.

## Supplementary information

supplementary legends

supplementary figures
