# Supplementary material for: KCTD15 inhibits the Hedgehog pathway in Medulloblastoma cells by increasing protein levels of the oncosuppressor KCASH2
Source: Oncogenesis. 2019 Nov 4;8(11):64. doi: 10.1038/s41389-019-0175-6 (PMC6828672; doi:10.1038/s41389-019-0175-6)
Supplement: Supplementary file 1 — supplementary legends [file 41389_2019_175_MOESM1_ESM.docx]

**Supplementary information**

**Supplementary figure legends**

**Supplementary Fig. 1. KCTD15 is the most represented interactor of KCASH2 in the Co-IP assay.**

**a** Schematic representation of the dual tagged KCASH2 vector used for Co-IP assay. **b** List of the most relevant KCASH2 interactors with corresponding numbers of unique peptides obtained from the MS analysis.

**Supplementary Fig. 2. KCTD15 and KCASH2 co-localize**. Immunofluorescence staining of MB DAOY cells transfected with vectors expressing KCTD15-Flag (in green) and KCASH2-HA (in red). Both proteins largely co-localize; KCASH2 (red) has a diffused localization (panel I); KCTD15 (green) has a similar localization although nuclear staining appears more intense than KCASH2 (II). Hoechst staining indicates nuclei (III); the last panel (IV) represents a merged figure.

**Supplementary Fig. 3.** **SHH-dependent MBs frequently present both KCTD15 and KCASH2 reduced expression.** **a-c** 47 human sporadic MBs belonging to different molecular groups have been assessed for KCTD15 (**a**) and KCASH2 (**b**) expression by Q-RT-PCR analysis. The relative mRNA levels were expressed as the ratio of the sample quantity to the mean values of 9 normal cerebella (Ctr). *P<0.05 G3 versus SHH, **P<0.01 G4 versus SHH (Mann-Whitney test). **c** No significant correlation was observed (r: 0,1; p= 0.69) between KCASH2 and KCTD15 expression analysis in SHH human MB.

**Supplementary Fig. 4. Generation of KCASH2 knock down HEK293T cells.** . Western blot analysis of KCASH2 protein expression in lentiviral control and KCASH2 shRNA-transduced HEK293T cells. Alpha-tubulin is shown as loading control.

**Supplementary Fig. 5. KCTD15 is not able to decrease GliRE luciferase activity in KCASH2 knock-down HEK293T**. Cells were transfected with 12× GliRE-Luc, Gli1, KCTD15. pRL-TK Renilla was cotransfected as a normalizer.

**Supplementary Fig. 6. KCTD15 overexpression reduced EdU incorporation in DAOY cells.** Dividing cells incorporate EdU (shown in green). Cells’ nuclei are counterstained with Hoechst (in blue).

**Supplementary Fig. 7. Overexpression of KCTD15 increases apoptosis in DAOY cells. a** DAOY cells were transfected with KCTD15 or empty vector (Ctr). The percentage of late apoptotic cells that incorporated both PI and Hoechst dyes was measured using the ImageJ software. Results are expressed as the mean ± SD of five independent experiments (**P<0.01 calculated by a Student’s t-test). **b** WB analysis of cleaved Caspase 3 protein in DAOY cells transfected as above. Actin is shown as loading control.

**Supplementary Fig. 8. KCASH2 and KCT15 do not localize in the primary cilium.** DAOY MB cells were transfected with HA-KCASH2 or Flag-KCTD15. Immunostaining was performed with anti ARL13B (red) to identify primary cilia and anti-tags to stain tagged KCASH2 or KCTD15 exogenous-proteins (green).
