# Supplementary material for: KCTD15 inhibits the Hedgehog pathway in Medulloblastoma cells by increasing protein levels of the oncosuppressor KCASH2
Source: Oncogenesis. 2019 Nov 4;8(11):64. doi: 10.1038/s41389-019-0175-6 (PMC6828672; doi:10.1038/s41389-019-0175-6)
Supplement: Supplementary file 2 — supplementary figures [file 41389_2019_175_MOESM2_ESM.pdf]

A

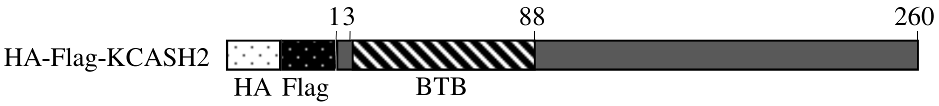

B

List of Interactors	Number of unique peptides
KCASH2	17
**KCTD15**	**13**
Isoform Long of Sodium/potassium-transporting ATPase subunit alpha-1 precursor (IPI00006482)	4
Polymeric immunoglobulin receptor precursor (IPI00004573)	4
Mitotic spindle assembly checkpoint protein MAD2 (IPI00012369)	4
Isoform 1 of Transcription intermediary factor 1-beta (IPI00438229)	4
Isoform 1 of Protein transport protein Sec61 subunit alpha (IPI00218466))	3
Isoform 1 of Melanoma-associated antigen D1 (IPI00328354)	3
Isoform 2 of Import inner membrane translocase subunit TIM50, mitochondrial precursor (IPI00418497)	3

Spiombi et al. Suppl. Figure 1.

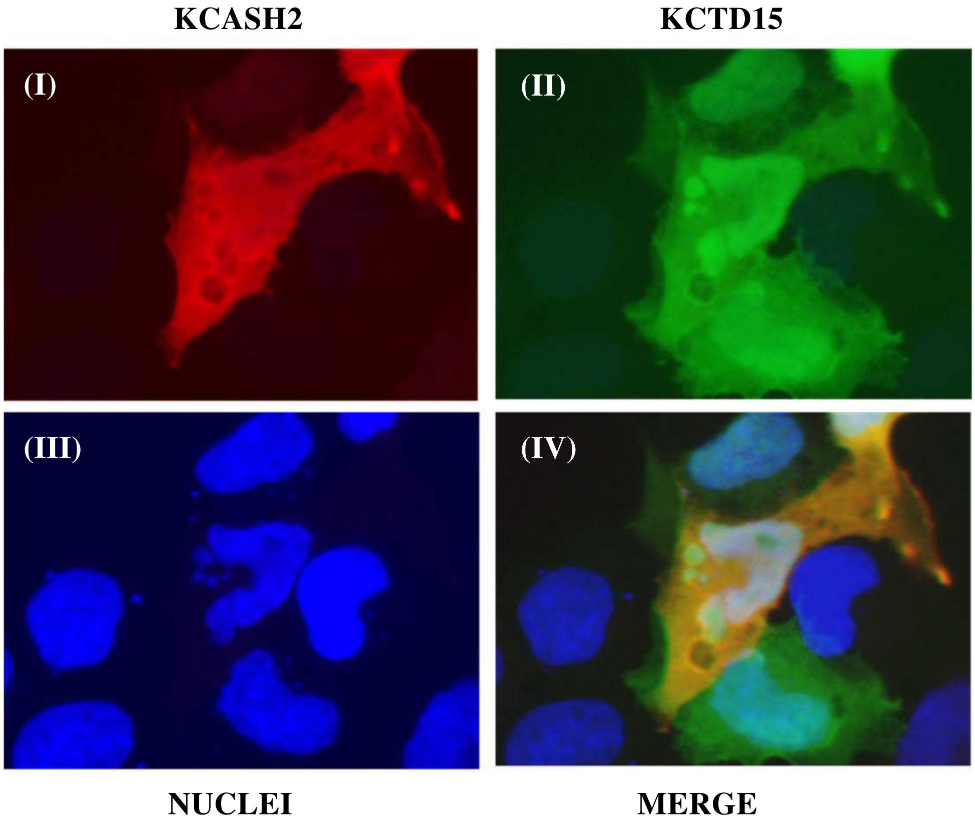

Spiombi et al. Suppl. Figure 2

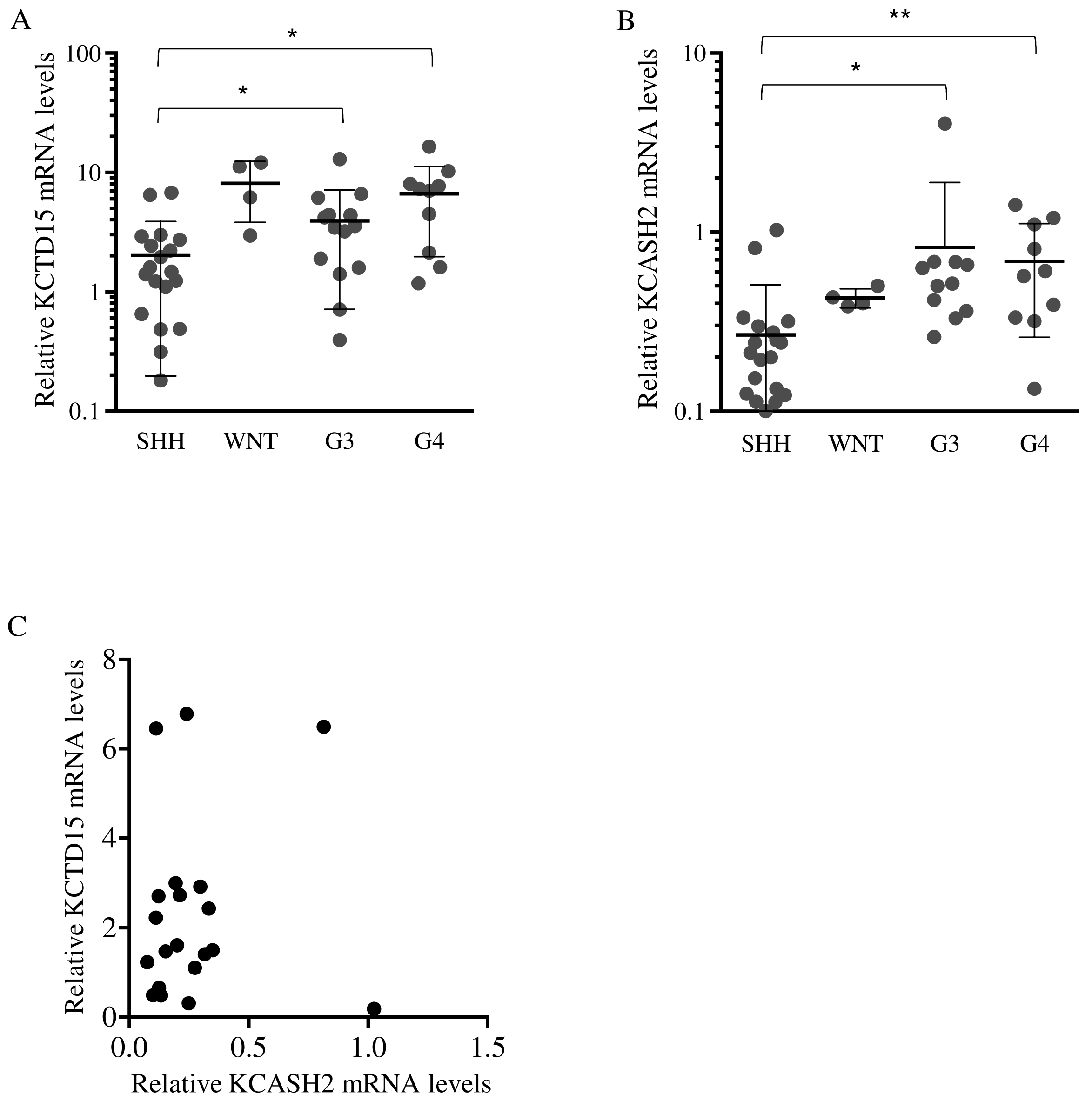

Spiombi et al. Suppl. Figure 3

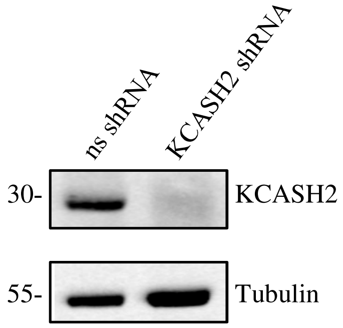

Spiombi et al. Suppl. Figure 4

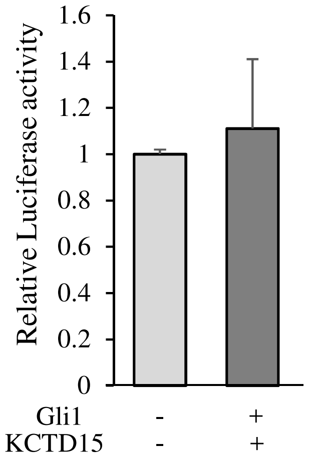

Spiombi et al. Suppl. Figure 5

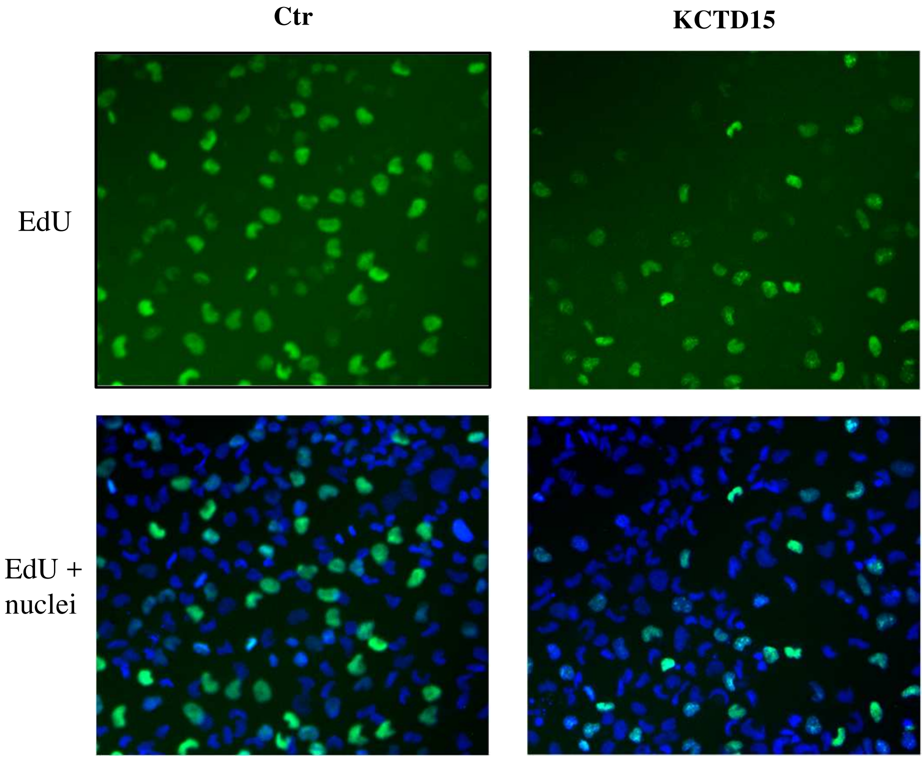

Spiombi et al. Suppl. Figure 6

A

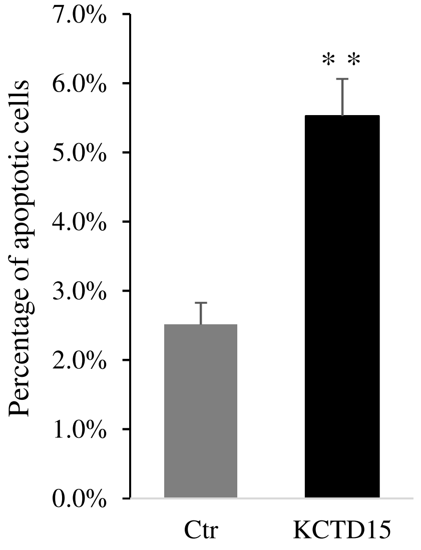

B

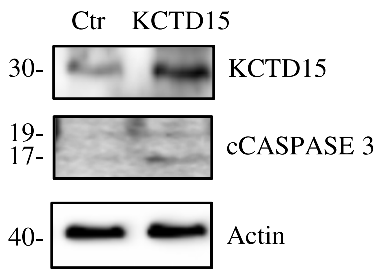

Spiombi et al. Suppl. Figure 7

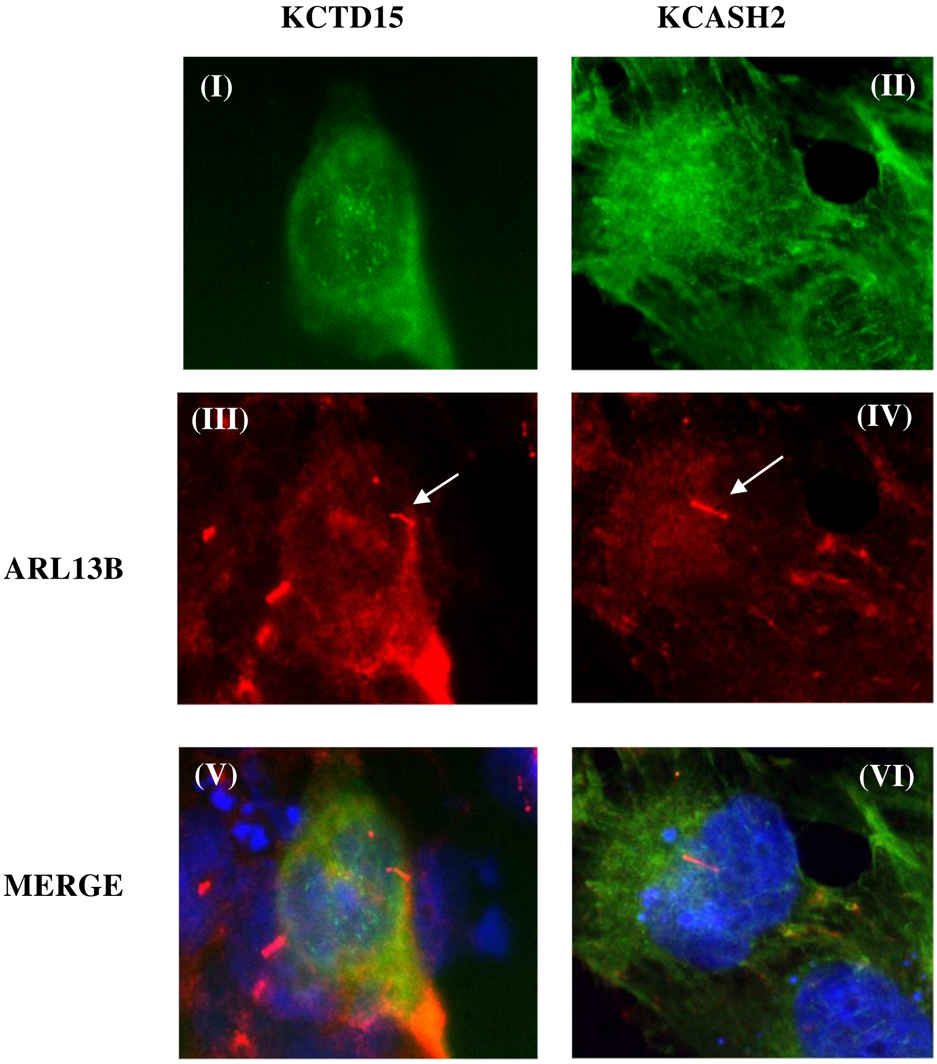

Spiombi et al. Suppl. Figure 8
